# From Human Cytogenetics to Human Chromosomics

**DOI:** 10.3390/ijms20040826

**Published:** 2019-02-14

**Authors:** Thomas Liehr

**Affiliations:** Jena University Hospital, Friedrich Schiller University, Institute of Human Genetics, Am Klinikum 1, D-07747 Jena, Germany; Thomas.Liehr@med.uni-jena.de; Tel.: +49-36451-9396850

**Keywords:** chromosomes, cytogenetics, chromosomics, interphase-architecture, epigenetics

## Abstract

Background: The concept of “chromosomics” was introduced by Prof. Uwe Claussen in 2005. Herein, the growing insights into human chromosome structure finally lead to a “chromosomic view” of the three-dimensional constitution and plasticity of genes in interphase nuclei are discussed. This review is dedicated to the memory of Prof. Uwe Claussen (30 April 1945–20 July 2008). Recent findings: Chromosomics is the study of chromosomes, their three-dimensional positioning in the interphase nucleus, the consequences from plasticity of chromosomal subregions and gene interactions, the influence of chromatin-modification-mediated events on cells, and even individuals, evolution, and disease. Progress achieved in recent years is summarized, including the detection of chromosome-chromosome-interactions which, if damaged, lead to malfunction and disease. However, chromosomics in the Human Genetics field is not progressing presently, as research interest has shifted from single cell to high throughput, genomic approaches. Conclusion: Chromosomics and its impact were predicted correctly in 2005 by Prof. Claussen. Although some progress was achieved, present reconsiderations of the role of the chromosome and the single cell in Human Genetic research are urgently necessary.

## 1. Introduction

Prof. Uwe Claussen (30 April 1945–20 July 2008) was a Human Geneticist and brilliant scientist with specific interest in cytogenetics and chromosome biology [1]. Thus, he was a rare representative of those scientists in Human Genetics field interested in the function of interphase and metaphase chromosomes. In 2002, he clearly demonstrated that chromosomes in these two stages are much more similar than generally accepted [2]. 

Prof. Claussen was full of ideas and had innumerous visions on what could or should be studied next and how this could be realized. Still, only 2% of his ideas were, as he conceded, realistic and worth investing more time into them. Typical of his nature, he introduced the concept of “chromosomics” in 2005 [3]. This concept was brought forth into a scientific environment already full of new “-omics” inventions [4,5,6,7,8,9,10,11,12]. 

**Figure 1:**
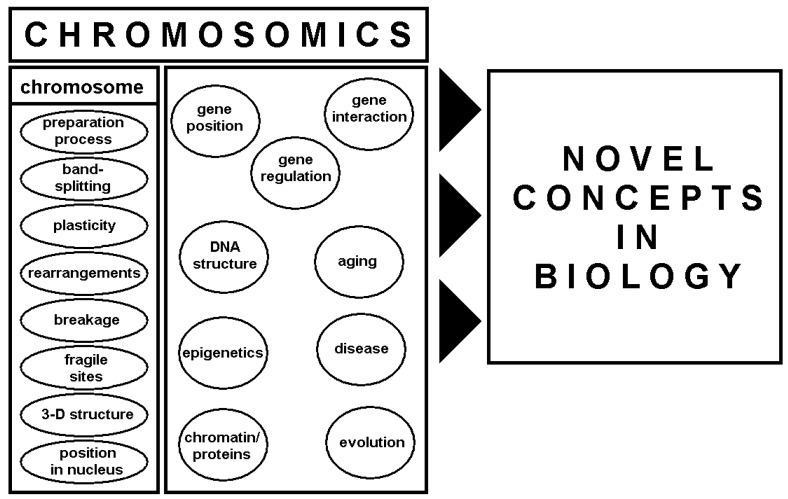
Schematic depiction of chromosomics-concept. Results from studies on chromosomes (first column) are combined with results from other studies (second column)—chromosomics encompasses all of this research. This combination leads to novel concepts in biology of, but not restricted to, chromosomes.

This review summarizes how insights into human chromosomes, primarily based on cytogenetic studies, finally advanced the chromosomics-concept (Figure 1) and led to acceptance in current human genomic oriented theories.

## 2. Human Chromosomes

Chromosomes, meaning nothing other than “stained bodies”, were visualized only after the development of light microscopy. However, this technological development was only able to deliver reliable and reproducible results by the late 1800s. Accordingly, anomalies of mitoses were described in 1879 by Julius Arnold [13], one year after Walther Flemming first introduced the terms “chromatin” and “mitosis” into the field [14]. Still, nine years passed before Wilhelm von Waldeyer-Hartz invented the term “chromosome” for the structures involved in mitosis [15]. 

Interestingly enough, Gregor Mendel already described chromosomes (without knowing) in 1866 in his law of independent assortment (his second law). There, he postulated “coupling groups.” “Coupling groups” describe the fact that some genes (leading to specific phenotypes) may be connected, and are normally inherited together, rather than separately. In other words, Mendel had theorized that two (or more) genes may be located on the same chromosome, and others were located on different “coupling groups,” or what we now know as chromosomes [16]. 

As reviewed elsewhere [17,18], during the following 80 years, further insights into human chromosomes were only very marginal, even though countless microscopic studies on the nucleus were completed and published by Walther Flemming [19] and others [13,20,21]. Strikingly, in 1903, Theodor Boveri [21] and Walter Sutton [22] published—in big parts correct—theories on the putative role of chromosomes in inheritance [22,23,24]. In 1914, Theodor Boveri [25] also wrote a small booklet that suggested a role for chromosomes in cancer, which again was largely correct. Four years earlier, in 1910, Thomas Hunt Morgan had shown in Drosophila, that genes are aligned on chromosomes like pearls on a necklace [26,27].

In 1952, one year before James Watson and Francis Crick published their seminal works on the structure of deoxyribonucleic acid (DNA) [28,29], Tao-Chiuh Hsu confirmed the suggestion of Theophilius Shickel Painter [30] that human, as other great apes, should carry 48 chromosomes [31]. Shortly thereafter, in 1956, this was disproven by Joe Hin Tijo and Albert Levan [32]. Once the correct modal human chromosome number was established as 46, the clinical applied human cytogenetics era finally burgeoned. Now, it was possible to correlate inherited and acquired human diseases with specific numerical and/or structural chromosomal aberrations (for review, see References [17,18]). In parallel, from the 1950s to 1970s, reproducible approaches for the cultivation of human cells, chromosome preparation, staining and banding were developed, ultimately refined and finally established for use in research and diagnostics (for review, see References [17,18]). In particular, the Q-banding (Quinacrine based banding of chromosomes) approach introduced by Lore Zech (Uppsala, Sweden) [33] in the 1970s [34] paved the way for the advancement of the field. This discovery led to hundreds of thousands of human chromosomal analyses performed every year worldwide, principally for diagnostics of inborn errors and cancer. 

With the advent of fluorescence in situ hybridization (FISH) in the 1980s (for review, see Reference [18]), diagnostic tests of human chromosomes surged into millions of analyses per year, facilitated by the rapid advancement of suppliers of commercial FISH-probes [35]. However, insights into the biology, three-dimensional structure and function of human chromosomes did not flourish in the same way. This can be attributed to the ethos of Human Genetics, being focused on the support of the individual patient rather than on chromosome research [36]. For the past 25 years, this focus was unwavering, as the advancing technical capabilities for studying the genetic basis of human diseases have grown and continue to progress tremendously [36]. Today, laboratories can detect disease causing copy number alterations by array comparative genomic hybridization (aCGH) (e.g. Reference [37]), small mutations including deletions and/or rearrangements by different polymerase chain reaction (PCR)-based approaches like MLPA (multiplex-ligation dependent probe amplification) (e.g. Reference [38]), uniparental disomy by microsatellite analyses or single nucleotide polymorphism (SNP)-based aCGH [39], and mutations by high-throughput techniques like next generation sequencing (e.g. Reference [40]). As most of these applications are neither single cell-directed nor chromosome-oriented, but involve analyses of DNA, chromosomes are unfortunately left out of the focus of Human genetic research [41]. Thus, research focused on the three-dimensional structure of chromosomes and interphase cells is the exception, rather than the rule, in Human Genetics institutions. This is regrettable, as FISH and so-called Hi-C (high-throughput sequencing based chromosome conformation capture techniques) approaches could enable insights into chromosome biology (e.g. Reference [42]), which Walther Flemming could not even dream about.

## 3. Contributions of Prof. Uwe Claussen to Human Cytogenetics 

Prof. Claussen had multiple clinical contributions (e.g. References [43,44,45,46,47,48,49,50,51]), but his research papers since 1980 focused mostly on chromosomes, their behavior, and, as it seems, questioning dogmas that might soon be refuted. His first work in the 1980s toward that aim included attempts to get a result from human amniocytic fluid faster than 2–4 weeks of cultivation. Although others had suggested that there was no way to do it faster, Prof. Claussen soon developed an approach to identify and collect mitotic cells from amniotic cell cultures by a pipette under microscopic control and succeeded in using these cells for rapid cytogenetic results [52]. Unfortunately, although this approach is also applicable to tumor cells [53], it is no longer used in practice. 

Prof. Claussen was particularly famous for his studies based on glass-needle chromosome microdissection (midi). He applied midi on banded and unbanded chromosomes for DNA-library construction. The latter were used in molecular genetic or molecular cytogenetic studies for gene-identification or chromosome staining, respectively [54,55,56,57]. Prof. Claussen, working with Nikolai Rubtsov (Novosibirsk, Russia), found that during the midi-process chromosomes are highly elastic and can be stretched [58]. What was initially just an interesting anecdotic finding termed chromosome-stretching by midi, soon turned out to reflect a feature of chromosomes most likely involved in an important role of their function in vivo. Prof. Claussen and his colleagues proved that chromosome spreading during cytogenetic preparation is similar to what occurs during chromosome-stretching [59]. In parallel, Uwe Claussen and his colleagues also demonstrated that it was possible to determine the chromosomal banding level of a metaphase simply by assessing a few chromosomal bands [60], and soon focused their efforts on breaking yet another dogma. 

Upon review of different banding schemes in the international system of cytogenetic (now called cytogenomic) nomenclature (ISCN) [61], readers are left with the impression that for each chromosome presented, band- and sub-band-nomenclature describes biological facts, e.g. that the band 6q21, being visible at a resolution of 300 bands per haploid genome, splits up into three sub-bands 6q21.1, 6q21.2, and 6q21.3 at the 850 band level. However, Prof. Claussen demonstrated via chromosome-stretching that the band 6q21 does not split up further but becomes what is called at 850 band level “sub-band 6q21.1”. Bands 6q21.2 and 6q21.3 (850 band level), on the other hand, derive in reality from band 6q22 (300 band level) [62]. In further studies of the X-chromosome [63] and later for all human chromosomes [64,65], this finding was confirmed to be a general rule: Giemsa-light bands never create new sub-bands; new sub-bands only evolve during chromosome-stretching (or when compared to less condensed chromosomes) from Giemsa-dark bands. Thus, ISCN provides only a nomenclature of the bands, but the biological band-splitting was discovered and described based on Prof. Claussen’s detection of chromosome-stretchability [65]. Furthermore, Prof. Claussen suggested this kind of band splitting reflects the protein-packing within chromosomal bands and that this packing may be one reason why genes in Giemsa-light bands are more accessible to transcription than G-banding by Trypsin and Giemsa (GTG-) dark bands. Together, these studies suggest that the chromosome parts which can be artificially stretched are also more flexible in the living nucleus and can be accessed much easier by the transcription-machinery.

By 2002, millions of cytogenetic preparations were completed worldwide. However, no one understood what really happened during the preparation—especially during the “air-drying-step.” That year, a “Claussen-typical” study was completed and published [66], which demonstrated how chromosome-spreading onto the slides really works. The paper included a clear outline of process and established that when a chromosome-suspension is dropped from 2 mm vs. 2 m on the slide surface, height makes no difference in preparations. Rather, it is the humidity of the air which is critical and impacts the length of chromosomes on the glass slide. 

Prof. Claussen also focused on the development of new approaches which could be applied in further chromosomal research. Together with Gabriele Senger and Ilse Chudoba, he suggested a midi-based FISH banding approach [67] completed in 2002 in Jena [68]. In addition, he put forth ideas that generated a single-copy-probe based FISH banding approach [69], centromere-specific multicolor-FISH (cenM-FISH) probe sets [70], a probe set capable of distinguishing parental chromosomes [71], and a new platform to perform FISH in three-dimensionally preserved interphase nuclei [72]. These new techniques and probe sets were later applied in studies of chromosome structure by Prof. Claussen and his colleagues focused on concepts within the field of “chromosomics” [3]. 

Several studies were completed between 2002 and 2008, largely using FISH-banding to learn more about interphase and chromosome-structures. Studies based on FISH-banding in interphase confirmed that chromosomes principally keep their size and do not get extended like spaghetti during completion of the cell cycle [2,73,74,75,76,77,78,79,80,81]. This finding remains in contradiction to most textbooks, which continue to incorrectly postulate that interphase chromosomes are completely decondensed. Yet again, another dogma was shattered through the work of Prof. Claussen and colleagues, confirmed in parallel by the work of Thomas Cremer and others [82,83,84,85,86,87]. 

## 4. Chromosomics 

Previous studies and thoughts mentioned herein led Prof. Claussen to propose the “chromosomics” concept in 2005 [3] (Figure 1). Shortly thereafter, the term “chromosomics” was also used in several different contexts from the original concept including two publications, seemingly without an awareness of the 2005 work defining this word [3]. One Russian paper combined the term into “comparative chromosomics” and used it in the context of comparative genome mapping in evolution research [88]. One US-American publication defined chromosomics as the application of a specific commercially available FISH-probe-set [89]. In addition, the term is currently used in other settings, including a Japanese company called Trans Chromsomics [90]. On Wiki, one can even find the term chromosomics summarized under systems biology, side-by-side with other -omics, such as genomics, transcriptomics, translatomics, proteomics, and more [91], a finding which would have been appreciated by Prof. Claussen. 

According to him, the term “chromosomics” was introduced “to draw attention to the three-dimensional morphological changes in chromosomes, that are essential elements in gene regulation” [3]. His idea was to subsume all chromosome-related research with the goal “to lead us to novel concepts in biology” under this term [3]. This is in contrast to other omics-designations, which aim to show importance of their fields by separating it from others [3]. Thus, chromosomics includes the following kinds of studies (Figure 1):

- “on plasticity of chromosomes in relation to the three-dimensional positions of genes, which affect cell function in a developmental and tissue-specific manner during the cell cycle” [3]. This included studies on chromosome structure in meta- and inter-phase [2,73,74,75,76,77,78,79,80,81], as well as studies by Thomas Cremer [82,83,84,85,86,87] and others [92,93,94,95,96,97,98] that used three-dimensional-FISH and HiC-analyses [42,99,100,101,102,103,104,105,106]. Today, it is theorized that gene expression is dependent upon and regulated by chromosome structure in interphase. Thus, new concepts are already being integrated into transcriptomic research, with chromatin modifications largely considered major epigenetic factors influencing gene expression [99].
- “into chromatin-modification-mediated changes in the architecture of chromosomes, which may influence the functions and lifespans of cells, tissues, organs and individuals.” Insights into the flexible three-dimensional structures of metaphase chromosomes may also help to understand the influence of aforementioned “positional effects” on cells at different stages of their development. One important consideration is the recently demonstrated fact that each cell of the human body remembers which of the homologous chromosome sets derives from the mother and father of the individual [81]. In addition, effects of copy number alterations that appear during aging and their effects on nuclear architecture have yet to be established and further studies are warranted [107].
- on “species-specific differences in the architecture of chromosomes, which has been overlooked in the past” [3]. In that sense, the use of the word chromosomics was correct as used the aforementioned Russian colleagues [88]. Chromosomics studies with evolution focus on the construction of the interphase stage and the effects of this architecture were already performed e.g. in different mammals, reptiles and other species [75,108,109,110,111]. It is still unknown if conserved genes in mammalians keep their position in the same kind of chromosomal band (Giemsa-dark or –light) during evolution. Further studies are needed to elucidate whether changes in position lead to differential expression.
- on “the occurrence and prevalence of chromosomal gaps and breaks and interchanges” [3]. The focus here includes fragile sites [112] and their putative role as seeding points of (i) evolutionary conserved breakpoints [112,113,114], (ii) breakpoints observed in inherited [112,115], and (iii) acquired chromosomal aberrations in tumors [112,116,117]. Recently, as originally suggested by Prof. Claussen [3], fragile site related breaks were attributed to chromosome three-dimensional structure and function rather than to DNA-sequence [118,119,120].

## 5. Conclusions: Chromosomes and Their Appreciation in Nowadays Human Genetics

Without regard to the aforementioned astonishing new insights into human chromosomes, the field of Human genetics remains mainly diagnostic oriented, and studies upon chromosomes continue to be called outdated. The latter can be observed in the annual meeting of European Human Genetic societies, which neither included studies on interphase architecture, nor on chromosomes biology in the agenda of any educational or concurrent session, satellite meeting, workshop, or poster session during the past 10 years, at the least [121].

In contrast, findings known in human cytogenetics for more than one or two decades, including chromosome fragmentation and reshuffling of genome parts [122,123]/pulverization [124,125,126] and gene amplification based on double minutes or homogeneously staining regions [127,128] were recently “rediscovered” as chromotripsis and published in the highest ranked available journals of the field [129,130,131]. This was especially shocking to people with a good knowledge in cytogenetics, although the rediscoveries provided a much better resolution level due to new high-throughput approaches than previously known, the fact remains that chromosome shattering is absolutely not a new finding in the field [122,123,124,125,126,127,128]. A similar excitement was observed after the finding of copy number variants (CNVs) of several megabasepair (Mb) within the human genome [132,133], which, for cytogeneticists, was not at all unexpected due to previously known cytogenetically visible and, thus, much larger CNVs [133]—both heterochromatic [133,134] and euchromatic [133,135].

In conclusion, a robust recollection of Human genetic research from its roots via a comprehensive understanding of human chromosomes would lead to a much better interpretation of diagnostic results based on sound knowledge of chromosome structure and function—i.e., through chromosomics (Figure 1). This is even more desirable, as other disciplines besides Human Genetics, like scientists in pediatrics, cancer research, and/or neurology see this potential, and do for example research on silencing of a third copy of chromosome 21 using CRISP/Cas9 system and XIST [136].

## Abbreviations

aCGH	array comparative genomic hybridization
CNVs	copy number variants
DNA	deoxyribonucleic acid
FISH	fluorescence in situ hybridization
GTG	G-banding by Trypsin and Giemsa
Hi-C	high-throughput sequencing based chromosome conformation capture techniques
ISCN	International System for Human Cytogenomic Nomenclature
Mb	megabasepair
MLPA	multiplex-ligation dependent probe amplification
PCR	polymerase chain reaction
Prof.	professor
Q-banding	Quinacrine based banding of chromosomes
